# Supplementary material for: Unveiling quantum phase transitions from traps in variational quantum algorithms
Source: npj Quantum Inf. 2025 Jun 4;11(1):93. doi: 10.1038/s41534-025-01038-5 (PMC12137133; doi:10.1038/s41534-025-01038-5)
Supplement: Supplementary file 1 — Supplementary information [file 41534_2025_1038_MOESM1_ESM.pdf]

Supplementary Information for "Unveiling quantum phase transitions from traps in variational quantum algorithms"

I. ROBUSTNESS OF VARIATIONAL PARAMETER OPTIMIZATION TO NOISE

In this section, we present a detailed numerical study of the impact of noise on the optimization of variational parameters in the 1D transverse-field Ising model (TFIM). To simulate realistic hardware conditions, each R_{ZZ} rotation gate in the variational circuit is immediately followed by two corresponding local depolarizing noise channels modeled by

$$\mathcal{N}_{\mathrm{DP}}(\rho) = \left(1 - \epsilon^{(\mathrm{DP})}\right)\rho + \epsilon^{(\mathrm{DP})}\frac{I}{4} \tag{S1}$$

with $\epsilon^{(\mathrm{DP})} = 0.01$ acting on corresponding qubits. We fix a shallow circuit with depth $p = 2$ and perform the optimization under two scenarios: one using an ideal (noiseless) circuit, and the other using the noisy circuit described above. In both cases, we compare the optimized parameters and the resulting quantum states to assess whether the presence of noise significantly alters the optimized variational parameters.

Fig. S1(a) presents a schematic that illustrates our expectation: as noise increases, the energy landscape becomes flatter, but the local minima remain nearly unchanged. Panels (b)–(d) then provide numerical results. In panel (b), we plot the relative distance between the ideally optimized rotation angles and those obtained from noisy optimization as a function of the transverse field g for two noise rates, $\epsilon^{(\mathrm{DP})} = 0.005$ and $\epsilon^{(\mathrm{DP})} = 0.01$. The relative distance is defined as

$$d_{\mathrm{rel}} = \frac{\sqrt{\|\boldsymbol{\gamma}_{\mathrm{noisy}} - \boldsymbol{\gamma}_{\mathrm{ideal}}\|_2^2 + \|\boldsymbol{\beta}_{\mathrm{noisy}} - \boldsymbol{\beta}_{\mathrm{ideal}}\|_2^2}}{\sqrt{\|\boldsymbol{\gamma}_{\mathrm{ideal}}\|_2^2 + \|\boldsymbol{\beta}_{\mathrm{ideal}}\|_2^2}}, \tag{S2}$$

where $\|\cdot\|_2$ is the Euclidean norm, which remains below 0.074 across all values of g. Panel (b) shows that when the optimized parameters (obtained from the noisy circuit) are embedded into an otherwise noiseless circuit with 14 qubits, the fidelity between the states produced by the noisy and ideal parameters remains high (all above 0.996) for both noise levels. In panel (c), we present detailed comparisons of 40 samples of rotation angles, demonstrating that the optimized angles obtained from noisy and noiseless circuits are in close agreement.

These results illustrate that moderate levels of local depolarizing noise have a minimal impact on the optimized variational parameters, thereby preserving the ability of our algorithm to accurately identify the effective order parameter and the critical point. Ref. [1] has shown that the inherent stochasticity of noise may help the quantum optimization process avoid strict saddle points. Although our study does not directly exploit this potential benefit, our findings of robust performance under moderate noise levels suggest that some amount of noise might even facilitate finding better local minima. We leave a detailed investigation of these effects for future work.

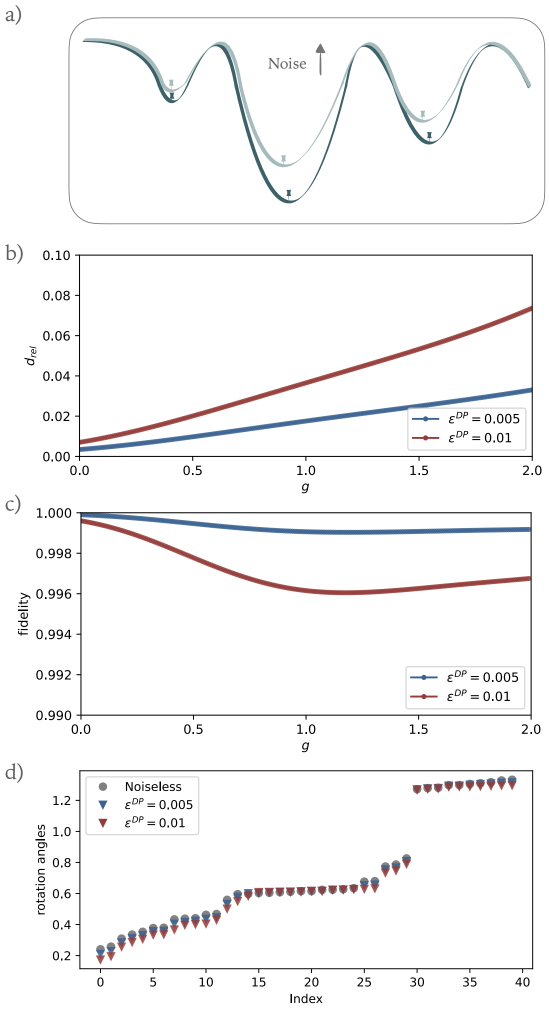

Figure S1. Robustness of optimized variational parameters in the 1D TFIM under local depolarizing noise. (a) A schematic illustration showing that, as noise increases, the energy landscape becomes flatter, while the positions of the local minima remain largely unaffected. (b) The relative distance d_{rel} between the ideally optimized rotation angles and those obtained from noisy optimization is plotted as a function of the transverse field g for two noise levels, $\epsilon^{(\mathrm{DP})} = 0.005$ (blue markers) and $\epsilon^{(\mathrm{DP})} = 0.01$ (red markers). (c) The state fidelity, computed by embedding the optimized parameters from both noisy and noiseless circuits into an ideal 14-qubit circuit, is plotted versus g for the same two noise levels. Fidelity values are consistently above 0.996, indicating minimal deviation. (d) A representative comparison of 40 sample sets of rotation angles from noisy and noiseless optimization.

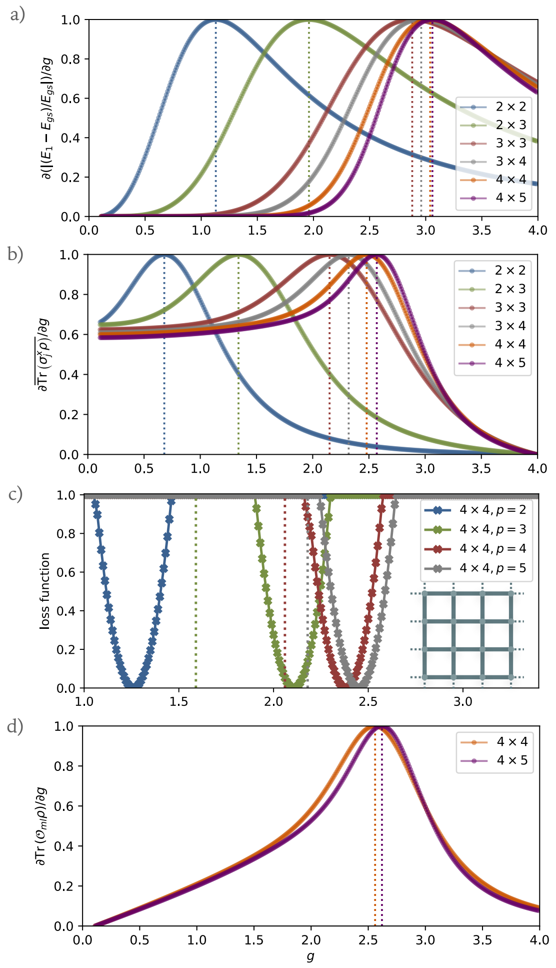

Figure S2. (a) Derivatives of the relative spectral gap as a function of the transverse field g for system sizes 2×2, 2×3, 3×3, 3×4, 4×4, and 4×5 under periodic boundary conditions. (b) Derivatives of the x-magnetization as a function of g for the same systems. (c) LASSO loss landscapes obtained from trapped states on a 4×4 grid with periodic boundary conditions for circuit depths $p = 2, 3, 4$, and 5. Dashed lines denote the critical boundaries estimated via x-magnetization. (d) Derivatives of the learned order parameter as a function of g for the ground state data of 4×4 and 4×5 grids. Dashed lines in panels (a), (b), and (d), denote estimated critical boundaries.

II. LARGER SYSTEM INVESTIGATIONS FOR THE 2D TFIM

In this section, we present additional numerical results for larger qubit grids with periodic boundary conditions. We consider several lattice configurations, including 2×2, 2×3, 3×3, 3×4, 4×4, and 4×5 systems. and analyse the estimation of the phase transition critical point using different metrics.

In panel (a) of Fig. S2, we plot the derivatives of the absolute relative spectral gap $(E_1 - E_{\rm gs})/|E_{\rm gs}|$ as a function of the transverse field g for the aforementioned system sizes. The estimated critical points from these derivatives are approximately 1.13, 1.96, 2.88, 2.96, 3.04, and 3.06 for the 2×2, 2×3, 3×3, 3×4, 4×4, and 4×5 systems, respectively. Notably, the last two estimates are very close to the thermodynamic limit value, $g_c = 3.04438$ [2, 3]. Panel (b) shows the derivatives of the x-magnetization as a function of g for the same set of system sizes. The corresponding estimated critical points are 0.68, 1.34, 2.15, 2.32, 2.48, and 2.57. These estimates are clearly less accurate than those obtained from the relative spectral gap, highlighting the limitations of local order parameters in finite systems.

We further implement our LASSO-based algorithm on a 4×4 grid with periodic boundary conditions for circuit depths $p = 2, 3, 4$, and 5. The average fidelities for these depths are 0.5473, 0.7336, 0.8674, and 0.9293, respectively. In panel (c) of Fig. S2, we display the LASSO loss landscapes for each circuit depth. The estimated critical points from the loss landscapes are 1.26, 2.11, 2.38, and 2.45 for $p = 2, 3, 4$, and 5, respectively. For comparison, we also include, using dashed lines, the critical boundaries obtained from the x-magnetization of the trapped states. Notably, for $p = 2$, the x-magnetization fails to provide an estimate, and for $p = 3, 4, 5$, the estimates derived from x-magnetization are considerably worse than those obtained via our algorithm. The dominant order parameter identified by our method in this case is $\mathcal{O}_{\rm ml} = \sum_{i,j} \sigma^x_{i,j}\sigma^x_{i+2,j+1}$.

Finally, in panel (d), we present the derivative of the learned order parameter (i.e., $\mathcal{O}_{\rm ml}$) with respect to g, computed from the ground state data for both the 4×4 and 4×5 grids. The derivative curves obtained using the learned order parameter yield more accurate estimates of the critical point (2.56 and 2.62, respectively) compared to those based on x-magnetization.

III. EXEMPLARY SET TRANSFORMER CODE AND COMPLEXITY ANALYSIS

In Fig. S3 we provide the exemplary Python code for the Set Transformer used in Section ??. Central to this architecture is the Self-Attention Block (SAB), which processes the input data through the mechanism of self-attention. In this setup, the normalized input, X_norm, serves simultaneously as the Query, Key, and Value. This configuration enables each element of the input feature interact with every other feature, facilitating a comprehensive internal representation that captures the underlying relationships.

In our proposed framework, a set of optimization grid points, whose total number we denote by N, is chosen as potential candidates for detecting phase transitions. For each valid center g, the algorithm collects a local dataset of size $2w$, formed by w parameter values below g (labeled -1) and w parameter values above g (labeled $+1$), and then trains a supervised model on these samples. Let $\ell = \ell(n)$ be the total number of observables (Pauli operators or otherwise) whose expectation values are measured

```
import torch.nn as nn


class SAB(nn.Module):
def __init__(self, d_model, num_heads):
super(SAB, self).__init__()
self.attention = nn.MultiheadAttention(d_model, num_heads)
self.norm = nn.LayerNorm(d_model)
self.dropout = nn.Dropout(0.1)

def forward(self, X):
X_norm = self.norm(X)
attention_output, _ = self.attention(X_norm, X_norm, X_norm)
attention_output = self.dropout(attention_output)
output = self.norm(X + attention_output)
return output

class SetTransformerRegressor(nn.Module):
def __init__(self, d_model, num_heads, num_layers, fc_intermediate_dim=16):
super(SetTransformerRegressor, self).__init__()
self.sab_blocks = nn.ModuleList([SAB(d_model, num_heads) for _ in range(
    num_layers)])
self.fc1 = nn.Linear(d_model, fc_intermediate_dim)
self.fc2 = nn.Linear(fc_intermediate_dim, 1)
self.activation = nn.ReLU()

def forward(self, src):
src = src.unsqueeze(1)
for sab in self.sab_blocks:
src = sab(src)
src = src.squeeze(1)
src = self.activation(self.fc1(src))
output = self.fc2(src).squeeze(-1)
return output
```

Figure S3. Exemplary code for the core architecture of the Set Transformer, utilized for detecting quantum phase transitions in the eSSH model. This includes implementations of both the Self-Attention Block (SAB) and the overall regression model structure.

and fed into the model. In practice, ℓ may be chosen to grow polynomially in the system size n if only local or few-body operators are included, or it may grow exponentially when higher-body correlations are considered.

In each training session, we fit a classical regression model—LASSO or a Transformer—to a labeled dataset of size $2w$. For LASSO, we employ a coordinate descent solver, which incurs a per-iteration computational cost of order $w\ell^2$. If the algorithm requires s iterations to converge, the cost of a single LASSO training run is $\mathcal{O}(s\,w\,\ell^2)$. When s and w remain nearly constant or grow only slowly with n, $\ell(n)^2$ becomes the dominating factor.

For Transformers inspired by the Set Transformer framework, the forward/backward pass of a single attention layer scales as $\mathcal{O}(\ell^2\,d)$, where d is the embedding dimension. In the presence of h attention heads, the overall cost for one layer is approximately $\mathcal{O}(h\,\ell^2\,d)$. If τ is the number of epochs or gradient updates, each Transformer training session has an overall cost of $\mathcal{O}(\tau\,h\,\ell^2\,d)$, possibly with additional constants from feedforward blocks. Because the dataset size is $2w$, τ generally stays modest in most practical settings, so again $\ell(n)^2$ often drives the scaling.

Since the sliding-window procedure trains a separate model at each center g, these costs must be multiplied by the number of centers N_c. If boundary effects in the parameter grid are negligible, N_c is of the same order as N. Consequently, applying LASSO at all window centers yields a total cost of $\mathcal{O}\big(N_c \times s\,w\,\ell^2\big)$, while a Transformer-based approach costs $\mathcal{O}\big(N_c \times \tau\,h\,\ell^2\,d\big)$. In typical setups, s, τ, h, d, and w are constants or grow only mildly with n, making $\ell(n)^2$ and N_c the primary contributors to the runtime. If $\ell(n)$ can be chosen to scale polynomially with n (or remain approximately constant), and N_c is a constant independent of n, then the overall complexity of the sliding-window detection algorithm scales polynomially with n. Under these assumptions, the protocol remains computationally feasible for moderate system sizes relevant to current or near-term quantum simulations.

Finally, we empirically evaluated the runtime of our

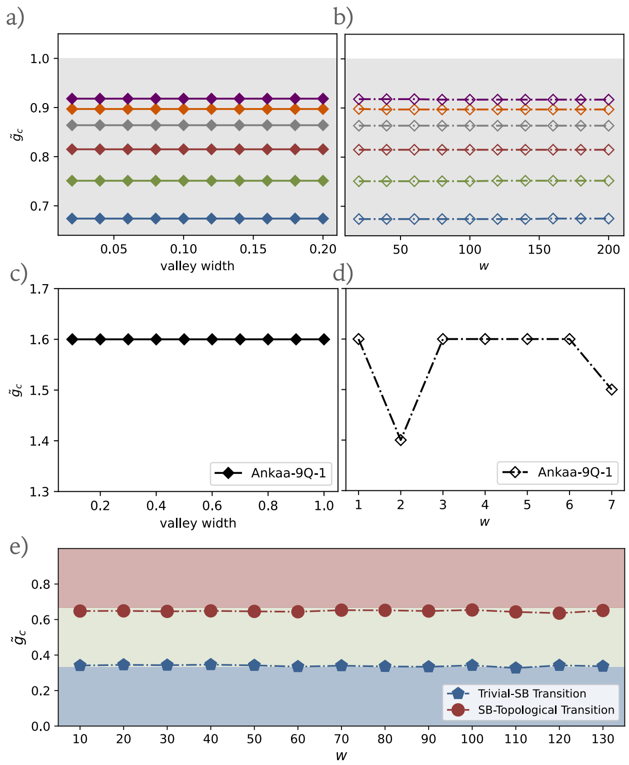

Figure S4. Predicted critical points for varying valley widths and window sizes, demonstrating the algorithm's robustness with respect to hyperparameters λ and w across diverse settings. (a) and (b) focus on processing numerical data for the 1D transverse-field Ising model (TFIM) using LASSO. In both panels, colours correspond to the different values of p defined in Fig. **??**(a, b). (c) and (d) present results from processing experimental data for the 2D TFIM using LASSO. (e) showcases the Set Transformer algorithm applied to the extended Su-Schrieffer-Heeger (eSSH) model with $\delta = 4$, highlighting its response to changes in w.

LASSO-based detection procedure on the 1D TFIM for system sizes $n = 4, 6, \ldots, 16$. As shown in Table S1, we fixed the window size at $w = 30$ and the number of sampled observables at $\ell = 30$, and scanned $N_c = 1940$ boundary points. Despite increasing n, the total runtime remains nearly constant at around 1–1.4 seconds, indicating that once w and ℓ are fixed, our post-processing overhead does not appreciably grow with the system size.

System size	4	6	8	10	12	14	16
Runtime (s)	1.381	1.395	1.308	1.405	1.381	1.296	1.405

Table S1. Total runtime (in seconds) for the LASSO-based detection of phase boundaries in the 1D TFIM, with $N_c = 1940$, $\ell = 30$, and $w = 30$ kept fixed, across various system sizes n.

From a theoretical standpoint, the iteration count or epoch number in these "black box" ML models depends largely on the complexity of distinguishing phases in feature space rather than simply on the system size. In many physically relevant models, this complexity does not necessarily scale with n. Consequently, even in regimes where a full classical simulation of the quantum state becomes intractable, the ML-based post-processing is expected remain tractable, provided it only requires distinguishing phases based on a polynomial number of measured features.

IV. ROBUSTNESS OF HYPERPARAMETER SENSITIVITY

This section evaluates the stability and robustness of our hybrid quantum optimization-machine learning algorithm against variations in two key hyperparameters: the regularization parameter λ within the LASSO algorithm, which inversely influences the width of the loss function landscape's valleys, and the window size w, crucial for both the LASSO and Transformer-based algorithms for setting the range of phase labels -1 and 1. It is essential to avoid excessively high values of λ, as this would result in all coefficients $\boldsymbol{\kappa}$ shrinking to zero, thereby reducing the LASSO's target cost function to a constant value of 1. We analyse the algorithm's performance using three illustrative examples: LASSO for the numerical data from the 1D TFIM, LASSO for the experimental data from the 2D TFIM, and the Set Transformer for the eSSH model with $\delta = 4$, where two phase transitions are observed.

The results, displayed in Fig. S4, include panels (a) and (b) for the 1D TFIM, panels (c) and (d) for the 2D TFIM, and panel (e) for the eSSH model. Panel (a) shows the predicted critical points for various valley widths with a constant window size of $w = 30$, demonstrating small sensitivity to this hyperparameter in LASSO as all lines remain constant. Panel (b) illustrates the predicted critical points across different window sizes with a set valley width of 0.06, where the maximum deviation observed is only 0.01. Panel (c) displays results for different valley widths at a window size of $w = 4$, consistently converging to 1.6. Panel (d) examines various window sizes at a valley width of 5, where the maximum variation is 0.2, but consistently approximates to the most likely value of 1.6. Panel (e) evaluates the Transformer's efficacy on the eSSH model with $\delta = 4$, considering various window sizes. It shows that the standard deviations associated with predictions of phase transitions from trivial to symmetry-broken (SB) phases and from SB to topological phases are 0.00525 and 0.00482, respectively, both indicating low variability.

These findings underline the limited sensitivity of our algorithm to changes in λ and w, confirming its robustness across different computational environments for both numerical and experimental data.

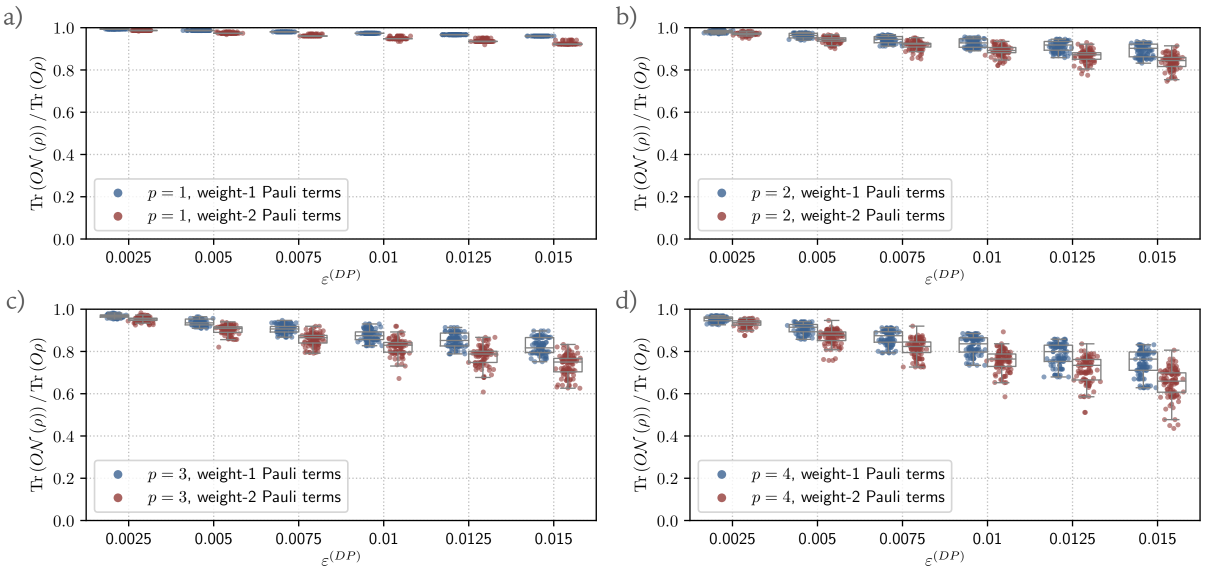

Figure S5. Effect of local depolarizing noise on Pauli expectation ratios in Hamiltonian variational ansatz circuits for the 1D TFIM. The four panels correspond to a different circuit depth: (a) $p = 1$, (b) $p = 2$, (c) $p = 3$, and (d) $p = 4$. For each panel, the horizontal axis represents the local depolarizing noise rate $\epsilon^{(\mathrm{DP})}$ and the vertical axis displays the ratio $R(O_j, \rho) = \mathrm{Tr}\,(O_j \mathcal{N}_{\mathrm{DP}}(\rho)) \,/\, \mathrm{Tr}\,(O_j\rho)$ for a randomly chosen Pauli operator O_j. Each data point is associated to the state ρ corresponding to one of 200 quantum circuits with a randomly sampled set of rotation angles $\{\boldsymbol{\beta}, \boldsymbol{\gamma}\}$. Blue points correspond to measurements of weight-1 Pauli operators, while red points correspond to weight-2 Pauli operators. The results demonstrate that for shallower circuits and lower noise rates, the variance of the ratio is minimal, validating the quasi-global depolarizing noise approximation for the randomly chosen observables.

V. ROBUSTNESS TO QUASI-GLOBAL DEPOLARIZING NOISE

Proof of Theorem ?? The feature vector $\boldsymbol{f}(g)$, input into the machine learning model, consists of the expectation values of Pauli operators from the set $\mathcal{P}_f$:

$$\boldsymbol{f}(g) = \left(\mathrm{Tr}\left(O_1\rho(\boldsymbol{\zeta}, \boldsymbol{\eta}; g)\right), \ldots, \mathrm{Tr}\left(O_\ell\rho(\boldsymbol{\zeta}, \boldsymbol{\eta}; g)\right)\right), \tag{S3}$$

where each O_j is a Pauli operator from the set $\mathcal{P}_f$.

Assuming the noise behaves like a global depolarizing channel Λ_ϵ, defined by:

$$\Lambda_\epsilon(\rho) = (1-\epsilon)\rho + \epsilon\frac{I}{2^n}, \tag{S4}$$

the expectation value of a Pauli operator O under this noise model becomes:

$$\mathrm{Tr}\left(O\Lambda_\epsilon(\rho(\boldsymbol{\zeta}, \boldsymbol{\eta}; g))\right) = (1-\epsilon)\,\mathrm{Tr}(O\rho(\boldsymbol{\zeta}, \boldsymbol{\eta}; g)) + \frac{\epsilon}{2^n}\,\mathrm{Tr}\left(O\right). \tag{S5}$$

Since the trace of non-identity Pauli operators is zero, this simplifies to:

$$\mathrm{Tr}\left(O\Lambda_\epsilon(\rho(\boldsymbol{\zeta}, \boldsymbol{\eta}; g))\right) = (1-\epsilon)\,\mathrm{Tr}(O\rho(\boldsymbol{\zeta}, \boldsymbol{\eta}; g)). \tag{S6}$$

Consequently, the noisy feature vector $\boldsymbol{f}_{\mathrm{noisy}}(g)$ is a scaled version of the original feature vector:

$$\boldsymbol{f}_{\mathrm{noisy}}(g) = (1-\epsilon)\boldsymbol{f}(g). \tag{S7}$$

When the noisy feature vector $\boldsymbol{f}_{\mathrm{noisy}}(g)$ is processed using LASSO, we can adjust the regularization coefficient λ by multiplying it by $(1-\epsilon)$. Considering the LASSO cost function as shown in Eq. (??):

$$\mathcal{C}(\boldsymbol{\kappa}, \lambda) = \left(\frac{1}{4w}\sum_{i=1}^{2w}\left(l_i - \kappa_0 - \sum_{j=1}^{\ell}\kappa_j f_{ij}\right)^2 + \lambda\sum_{j=1}^{\ell}|\kappa_j|\right) \tag{S8}$$

for the coefficients $\boldsymbol{\kappa} = (\kappa_0, \kappa_1, \kappa_2, \ldots, \kappa_\ell)$, the same cost function $\mathcal{C}$ can be achieved with the input $\boldsymbol{f}(g)$ and the adjusted coefficients:

$$\boldsymbol{\kappa}_{\mathrm{noisy}} = \left(\kappa_0, \frac{\kappa_1}{1-\epsilon}, \frac{\kappa_2}{1-\epsilon}, \ldots, \frac{\kappa_\ell}{1-\epsilon}\right). \tag{S9}$$

Thus, with adequate optimization, the classical loss landscape and the predicted critical points remain unchanged as long as we scale the regularization parameter λ by multiplying it with the factor $(1-\epsilon)$.

When processing the noisy feature vector $\boldsymbol{f}_{\mathrm{noisy}}(g)$ through the Transformer model, the initial step involves normalization of feature vectors. This step rescales data and effectively mitigating the scaling introduced by the noise, ensuring that the learning model's output remains unaffected by the scaling effect of the quasi-global depolarizing noise. ■

To empirically validate the conditions specified in Theorem ??, we conducted numerical simulations using the

Hamiltonian variational ansatz designed for the one-dimensional transverse-field Ising model (1D TFIM). In our simulation framework, each two-qubit rotation gate is subject to local depolarizing noise with a rate of $\epsilon^{(\mathrm{DP})}$, which results in an effective two-qubit error rate of approximately $2\epsilon^{(\mathrm{DP})}$.

We generated a representative ensemble of circuit outputs by randomly sampling 200 distinct sets of rotation angles $\{\boldsymbol{\beta}, \boldsymbol{\gamma}\}$. For each resultant quantum state ρ, we randomly select one Pauli operator O_j from the set of Pauli operators with weight 1 or 2. To ensure that only significant contributions are considered, we imposed the condition $|\operatorname{Tr}(O_j\rho)| > 0.2$. This thresholding procedure serves to filter out Pauli terms with negligible expectation values, which would otherwise be disproportionately affected by small noise-induced fluctuations.

The key metric we analyse is the ratio of the expectation value of the noisy state to that of the ideal (noiseless) state, defined as $R(O_j, \rho) = \operatorname{Tr}(O_j\mathcal{N}(\rho)) \,/\, \operatorname{Tr}(O_j\rho)$. We evaluated this ratio for a system size of $n = 10$ qubits and varying circuit depths $p = 1, 2, 3, 4$, while systematically adjusting the gate noise rate to different values $\epsilon^{(\mathrm{DP})} = 0.0025, 0.005, 0.0075, 0.01, 0.0125, 0.015$. The results, depicted in Fig. S5, display the distribution of the computed ratios for different configurations. Each subplot corresponds to a specific circuit depth, with blue markers representing ratios for weight-1 Pauli operators and red markers representing ratios for weight-2 Pauli operators.

We observe that the variance of the computed ratios is smaller when: (i) the circuit depth is low, (ii) the noise rate is small, and (iii) the observables are low-weight. The third behavior can be understood theoretically by noting that, for shallow circuits, the noise affecting individual qubits can be treated as statistically independent. For an operator that acts nontrivially on k qubits, the overall noise-induced attenuation is approximately given by the product $(1-\epsilon')^k$ for certain ϵ'. For low-weight operators (small k), the expectation values are only marginally reduced and exhibit low variance. In contrast, for high-weight operators, the multiplicative noise effects compound, leading to significantly lower expectation values and higher relative variance. As the circuit depth increases, the variance in the ratios grows, indicating that the quasi-global depolarizing approximation underlying Theorem **??** becomes progressively less valid. These results imply that by carefully selecting circuit depths and favoring lower-weight Pauli operators for $\mathcal{P}_f$, the impact of noise can be more effectively mitigated in practical implementations.

[1] J. Liu, F. Wilde, A. A. Mele, L. Jiang, and J. Eisert, Stochastic noise can be helpful for variational quantum algorithms (2023), arXiv:2210.06723 [quant-ph].

[2] H. W. J. Blöte and Y. Deng, Cluster Monte Carlo simulation of the transverse Ising model, Phys. Rev. E **66**, 066110 (2002).

[3] M. Schmitt, M. M. Rams, J. Dziarmaga, M. Heyl, and W. H. Zurek, Quantum phase transition dynamics in the two-dimensional transverse-field Ising model, Sci. Adv. **8**, eabl6850 (2022).
